# Theoretical Study on the Formation Mechanism of Ti(C,N) Inclusions and Titanium Content Control in High-Grade Non-Oriented Silicon Steel

**DOI:** 10.3390/ma19091684

**Published:** 2026-04-22

**Authors:** Jinwen Liu, Chuanmin Li, Fuqiang Zhou, Ben Zhang, Shanguo Du, Haiyan Tang, Jiaquan Zhang

**Affiliations:** 1 Capital Engineering & Research Incorporation Limited, Beijing 100176, China; lichuanmin@ceri.com.cn (C.L.); zhoufuqiang@ceri.com.cn (F.Z.); zhangben@ceri.com.cn (B.Z.); dushanguo@ceri.com.cn (S.D.); 2 School of Metallurgical and Ecological Engineering, University of Science and Technology Beijing, Beijing 100083, China; tanghaiyan@metall.ustb.edu.cn (H.T.); jqzhang@metall.ustb.edu.cn (J.Z.)

**Keywords:** high-grade non-oriented silicon steel, Ti(C,N) inclusions, slag carryover, TiO_2_ content, ESP

## Abstract

High-grade non-oriented silicon steel is a critical material for new energy vehicles and energy-efficient appliances due to its superior magnetic properties. However, these properties are significantly degraded by non-metallic inclusions, particularly Ti(C,N). This study employs integrated thermodynamic and kinetic calculations to systematically analyze the formation and growth mechanisms of Ti(C,N) inclusions in high-grade non-oriented silicon steel, trace the sources of [Ti], and propose targeted theoretical control strategies. Results indicate that Ti(C,N) inclusions do not precipitate above the liquidus temperature (1779 K). During solidification, microsegregation enriches Ti, C, and N; however, only TiN precipitates in the final stage as its ion product exceeds the solubility limit, whereas TiC remains undersaturated—findings valid within the present composition window and modeling framework. Inclusion size is governed by cooling rate and initial Ti/N content, where higher cooling rates yield finer inclusions and lower Ti/N content suppresses precipitation. Titanium originates from primary sources (raw materials and alloys) and secondary sources (decomposition or reduction of TiO_2_ in slag/refractories). Therefore, mitigating [Ti] requires strictly limiting primary input and suppressing secondary formation through optimized process control, such as reducing BOF slag carryover, lowering refining temperature, and controlling [Al] content.

## 1. Introduction

Driven by the “Dual Carbon” strategic initiative, the rapid expansion of the new energy vehicle (NEV) industry and the energy-efficiency transformation of the home appliance sector have increased the demand for high-grade non-oriented silicon steel. Distinguished by its low iron loss, high magnetic flux density, minimal magnetostriction, and superior magnetic isotropy, this material has become the cornerstone for motor manufacturing in these sectors [1,2,3,4].

However, non-metallic inclusions significantly degrade the magnetic properties of high-grade non-oriented silicon steel, with the extent of this deterioration governed primarily by inclusion size, followed by quantity and morphology [5,6,7,8,9,10]. Notably, when inclusion dimensions approach the width of magnetic domain walls, their pinning effect significantly exacerbates coercivity and hysteresis loss. Specifically, coercivity is known to scale inversely with inclusion size while exhibiting a direct relationship with inclusion density [11,12,13,14]. While coarse inclusions primarily compromise surface integrity and machinability, fine precipitates (<1 μm)—such as Cu_2_S, AlN, Ti(C,N), and MnS—exert a more detrimental influence on magnetic properties [15,16,17,18,19,20,21,22]. These fine particles pin grain boundaries, inhibiting grain growth and consequently deteriorating the magnetic efficiency of the steel.

Titanium-bearing secondary phases predominantly exist as TiC, TiN, or their solid solution, Ti(C,N) [23,24]. Given the identical NaCl-type crystal structure and negligible lattice mismatch between TiN and TiC, they readily form a continuous solid solution denoted as Ti(C_x_N_1−x_), where x represents the carbon molar fraction. The characteristics of Ti(C,N) shift from TiN-like to TiC-like as the C/N ratio increases. Thermodynamically, Ti exhibits a strong affinity for N, precipitating preferentially as TiN; therefore, TiC forms only after the Ti content exceeds the stoichiometric requirement for TiN formation [25]. Characterized by a high melting point and extreme hardness, TiN inclusions act as rigid obstacles [26]. Extensive thermodynamic and kinetic studies [27,28,29,30,31,32,33,34] reveal that TiN rarely forms in homogeneous high-temperature melts; instead, it precipitates during solidification when microsegregation elevates the local activity product of [%Ti] × [%N] beyond the saturation threshold. Consequently, control strategies in bearing and gear steels focus on restricting initial N and Ti levels [35,36,37,38] and optimizing cooling protocols [39,40,41,42] to mitigate TiN precipitation and refine particle size [43].

Compared to conventional hot rolling, the Endless Strip Processing (ESP) route offers distinct advantages, including a compact workflow, reduced capital investment, shorter lead times, significant energy savings, higher yields, and superior dimensional uniformity [44,45,46,47,48]. Crucially, the solidification time of ESP thin slabs is merely 1/10 to 1/5 of that of traditional thick slabs. This rapid solidification mitigates macrosegregation and internal cracking, creating favorable conditions for producing high-grade non-oriented silicon steel. Nevertheless, ESP presents unique challenges for silicon steel production: (1) the ultra-short workflow precludes post-casting composition adjustment or inclusion removal, necessitating single-batch delivery of ultra-pure, compositionally precise, and thermally stable molten steel; (2) high casting speeds (4–5 × conventional rates) hinder inclusion flotation in the mold, increasing the risk of nozzle clogging; (3) intense cooling promotes extensive columnar grain growth, predisposing the slab to surface undulations (alligatoring) after cold rolling; (4) the finer dispersion of second-phase precipitates in ESP sheets, compared to conventional products, can elevate iron loss and degrade final magnetic performance.

In view of the detrimental impact of fine Ti(C,N) inclusions on the magnetic properties of high-grade non-oriented silicon steel, this study employs integrated thermodynamic and kinetic calculations to systematically investigate the formation and growth mechanisms of Ti(C,N) and analyze relevant influencing factors. Furthermore, by tracing the sources of titanium in the steel and proposing corresponding control measures for [Ti] content, this work aims to provide theoretical guidance for controlling Ti(C,N) inclusions during the production of high-grade non-oriented silicon steel via ESP.

## 2. Thermodynamic and Kinetic Conditions for Ti(C,N) Precipitation

### 2.1. The Chemical Composition of High-Grade Non-Oriented Silicon Steel and Temperature of Solidus and Liquidus Line

The chemical composition of the high-grade non-oriented silicon steel used in this study (supplied by by Shougang steel (Qianan, China)) is presented in Table 1.

The liquidus (*T*_L_) and solidus (*T*_S_) temperatures of the high-grade non-oriented silicon steel were calculated using Equations (1) and (2), respectively [32].

(1) $$\begin{array}{l} T_{L}=1809-83w[\%C]-31.5w[\%S]-32w[\%P]\\ -5w([\%\mathrm{Mn}]+[\%\mathrm{Cu}])-7.8w[\%\mathrm{Si}]-3.6w[\%\mathrm{Al}]\\ -1.5w[\%\mathrm{Cr}]-2w[\%\mathrm{Mo}]-4w[\%\mathrm{Ni}]-18w[\%\mathrm{Ti}]-2w[\%V] \end{array}$$

(2) $$\begin{array}{l} T_{S}=1809-344w[\%C]-183.5w[\%S]-124.5w[\%P]-6.8w[\%\mathrm{Mn}]\\ -12.3w[\%\mathrm{Si}]-4.1w[\%\mathrm{Al}]-1.4w[\%\mathrm{Cr}]-4.3w[\%\mathrm{Ni}] \end{array}$$

According to the above calculation, the *T_L_* and *T_S_* of high-grade non-oriented silicon steel are 1779 K and 1761 K, respectively.

### 2.2. Thermodynamic Analysis of Ti(C,N) Precipitation Equilibrium

The chemical equations for the formation of Ti(C,N) are shown in Equations (3) and (4) [33], which are as follows:

(3) $$\left[\mathrm{Ti}\right]+\left[N\right]=\mathrm{TiN}(s)\;\Delta G_{\mathrm{TiN}}^{\theta }=-291000+107.91T$$

(4) $$\left[\mathrm{Ti}\right]+\left[C\right]=\mathrm{TiC}(s)\;\Delta G_{\mathrm{TiC}}^{\theta }=-157524+88.362T$$

(5) $$\begin{array}{l} \Delta G_{\mathrm{Ti}(C,N)}^{\theta }=-RTK_{\mathrm{Ti}(C,N)}^{\theta }=-RT\frac{a_{\mathrm{Ti}(C,N)}}{a_{\left[\mathrm{Ti}\right]}\times a_{\left[C,N\right]}}\\ =-RT\frac{1}{f_{\mathrm{Ti}}\times w[\%\mathrm{Ti}]\times f_{\left(C,N\right)}\times w[\%C,\%N]} \end{array}$$

(6) $$\mathrm{lg}f_{i}=\sum e_{i}^{j}\times w[\%j]$$

where Δ*G^θ^* is the standard Gibbs free energy of formation; *T* is the thermodynamic temperature; *K^θ^*_Ti(C,N)_ is the equilibrium constant; *a*_[Ti(C,N)]_, *a*_[Ti]_ and *a*_[C,N]_ represent the activities of Ti(C,N), [Ti] and [C,N], respectively; *f*_Ti_ and *f*_(C,N)_ denote the activity coefficients of Ti and (C,N); *w*[%Ti], *w*[%C,%N] and *w*[%*j*] are the mass percentages of Ti, (C,N) and *j* in steel; $e_{i}^{j}$ is the interaction coefficient of *j* on *i* in molten steel, as shown in Table 2; and *i* and *j* represent different components, respectively.

Then,

(7) $$\mathrm{lg}K_{\mathrm{TiN}}=\mathrm{lg}(w[\%\mathrm{Ti}]\times w[\%N])=-\frac{15198.09}{T}+5.3612$$

(8) $$\mathrm{lg}K_{\mathrm{TiC}}=\mathrm{lg}(w[\%\mathrm{Ti}]\times w[\%C])=-\frac{8227.02}{T}+4.1625$$

where *K* denotes the equilibrium concentration product of Ti and (C,N) required for Ti(C,N) precipitation. Equations (7) and (8) reveal that the thermodynamic criteria for Ti(C,N) formation are predominantly governed by the molten steel temperature.

**Figure 1:**
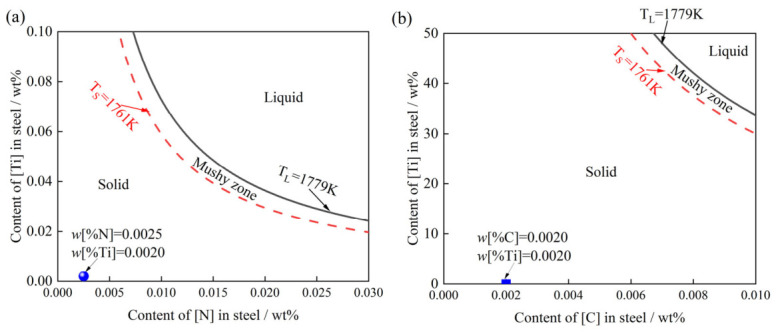
(**a**) N-Ti and (**b**) C-Ti equilibrium diagrams.

Figure 1 illustrates the equilibrium relationships for the N–Ti and C–Ti systems in high-grade non-oriented silicon steel across varying temperatures. As depicted, when the temperature exceeds the liquidus point (1779 K), the thermodynamic criteria for Ti(C,N) precipitation are not met. Consequently, Ti(C,N) inclusions are thermodynamically unstable and cannot persist in the molten steel. Given the specific chemical composition of the current steel grade, both TiN and TiC are positioned within the solid-phase region (Figure 1a,b). This indicates that TiN and TiC cannot precipitate from the liquid steel in this non-oriented silicon steel under thermodynamic equilibrium conditions.

The preceding analysis did not account for microsegregation phenomena during solidification. However, driven by microsegregation, the solute concentration in the residual liquid phase evolves continuously throughout the solidification process. Once the thermodynamic criteria for Ti(C,N) precipitation are satisfied at the solidification front, these inclusions nucleate and grow as cooling proceeds. The following section outlines the mathematical framework developed to analyze microsegregation under the context of ESP. To establish a focused scope, the model explicitly neglects fluid flow, macrosegregation, and non-equilibrium effects beyond its inherent assumptions. By concentrating on solute redistribution within interdendritic liquid pools, the analysis prioritizes diffusion-dominated transport at the microscopic scale. Consequently, the influence of fluid flow—responsible for macrosegregation over larger domains—is deemed negligible for the local equilibrium calculations.

### 2.3. Solute Segregation of Ti, C, and N During Solidification

#### 2.3.1. Microsegregation Models

Microsegregation during solidification arises from solute redistribution, driven by the difference in equilibrium solubility between the coexisting liquid and solid phases in the mushy zone, which leads to the continuous rejection of solutes into the liquid. The short solidification times and low diffusion coefficients preclude sufficient solute diffusion in the solid phase, inhibiting compositional homogenization at the end of solidification. To describe this phenomenon, various approximate models have been formulated based on the extent of solid-state diffusion [34,51]. These models generally postulate local thermodynamic equilibrium at the solid–liquid interface and perfect mixing within the liquid phase. Accordingly, a wide range of analytical equations incorporating distinct assumptions and simplifications are utilized to predict solute redistribution and associated phenomena.

Predicated on the assumption of equilibrium solidification, the Lever rule [34,51] postulates complete solute diffusion in both solid and liquid phases, rendering it suitable for interstitial elements with high diffusivity (e.g., C and N). Nevertheless, the validity of this model declines in the final stages of solidification. This limitation stems from the retarded diffusion rates within the solid matrix, a condition that is especially critical for larger substitutional solutes, such as manganese and titanium. Neglecting fluid flow, macrosegregation, and non-equilibrium solidification effects, the evolution of (C, N) concentrations at the solidification front as a function of solid fraction (fs) is described by the following expressions [34,42,52]:

(9) $$w{\left[\%C,\%N\right]}_{L}=\frac{w{\left[\%C,\%N\right]}_{0}}{1-(1-k_{\left(C,N\right)})\times f_{s}}$$

where *w*[%C,%N]_L_ represents the actual mass fractions of (C,N) in the liquid phase at the solidification front; *w*[%C,%N]_0_ denotes the initial mass fractions of (C,N) in molten steel; *k* is the partition coefficient, as shown in Table 3; and *f_s_* is the solidification fraction.

The Scheil model [51] postulates negligible diffusion in the solid, perfect mixing in the liquid, and local thermodynamic equilibrium at the interface, as shown in Equation (10). However, its validity is limited and does not cover the entire solidification range. In the final stages of solidification, the predicted liquid concentration diverges towards infinity—a result that is physically unrealistic. Consequently, application of the Scheil equation is generally restricted to rapid solidification scenarios.

(10) $$w{\left[i\%\right]}_{L}=w{\left[i\%\right]}_{0}{\left(1-f_{s}\right)}^{\left(k_{i}-1\right)}$$

However, the assumption of negligible diffusion in the solid phase is often considered unrealistic. To accurately predict microsegregation during metal solidification, particularly in industrial casting processes, it is essential to account for finite non-zero diffusion in the solid phase. Brody and Flemings [27,51] were the first to propose a model incorporating this factor, as shown in Equation (11). Their model assumes complete diffusion in the liquid and incomplete back-diffusion in the solid. Additionally, it postulates a fixed dendrite arm spacing, constant physical properties, local thermodynamic equilibrium at the solid–liquid interface, and linear liquidus and solidus lines on the equilibrium phase diagram.

(11) $$w{\left[\%i\right]}_{L}=w{\left[\%i\right]}_{0}{\left[1-(1-2\alpha_{i}k_{i})f_{s}\right]}^{\frac{k_{i}-1}{\left(1-2\alpha_{i}k_{i}\right)}}$$

(12) $$\alpha_{i}=\frac{D_{s,i}\tau }{{\left(0.5\lambda_{s}\right)}^{2}}$$

(13) $$\tau =\frac{T_{L}-T_{S}}{R_{c}}$$

(14) $$\begin{array}{l} \lambda_{s}=(169.1-720.9w[\%C])\times R_{c}^{-0.4955},0<w[\%C]\leq 0.15\\ \lambda_{s}=143.9R_{c}^{-0.3616}\times w[\%C]{)}^{\left(0.5501-1.996C_{0}\right)},0<w[\%C]\leq 0.15 \end{array}$$

where *w*[%*i*]_L_ represents the actual mass fractions of *i* in the liquid phase at the solidification front; *w*[%*i*]_0_ denotes the initial mass fractions of *i* in molten steel; α*_i_* is a back-diffusion parameter; *D*_s*,i*_ is the diffusion coefficient of the solute element *i* in the solid phase (cm^2^/s); *τ* is the local solidification time (s); λ_s_ is the secondary dendrite arm spacing (μm); and *R*_c_ is the cooling rate (K/s).

Studies [53] indicate that conventional slab continuous casting yields secondary dendrite arm spacings (SDASs) of 200–500 μm, corresponding to a cooling rate of approximately 0.5 K/s (at SDAS = 200 μm). In contrast, thin slab casting (ESP) significantly refines the microstructure, with SDAS values predominantly ranging from 52 to 180 μm (average ~99 μm), corresponding to a cooling rate of ~5 K/s. Consequently, this cooling rate was also employed as the primary basis for the subsequent calculations.

While the Brody and Flemings model converges to the Scheil equation in the asymptotic limit of α*_i_* → 0, it diverges from the Lever rule as α*_i_* → ∞.

Subsequently, the Clyne–Kurz model [54] defined a function *Ω_i_*(α*_i_*) (as given in Equation (15)) to substitute the back-diffusion parameter used by Brody and Flemings [27,51], which is as follows:

(15) $$\Omega_{i}(\alpha_{i})=\alpha_{i}(1-e^{-\frac{1}{\alpha_{i}}})-\frac{1}{2}e^{-\frac{1}{2\alpha_{i}}}$$

As α*_i_* approaches infinity, *Ω_i_*(α*_i_*) converges to 1/2, representing the limit of complete diffusion; under this condition, Equation (15) simplifies to Equation (9). This asymptotic behavior can be derived from the infinite series expansion of exp(x) = 1 + x + …. Conversely, as α*_i_* approaches zero, *Ω_i_*(α*_i_*) tends toward α*_i_* and subsequently vanishes. Consequently, Equation (15) reduces first to Equation (11) and ultimately to Equation (10). Therefore, Equation (15) provides a generalized framework that encompasses the entire spectrum of solid-phase mixing—from complete mixing to no mixing—making the model more representative of realistic solidification scenarios. Under the assumptions of limited solid-state diffusion and perfect liquid mixing, this approach demonstrates enhanced applicability for characterizing microsegregation in low-carbon steels.

Consequently, neglecting fluid flow, macrosegregation, and non-equilibrium solidification effects beyond the scope of the Clyne–Kurz model, the evolution of titanium concentration at the solidification front as a function of *f_s_* is described by the following expression [27]:

(16) $$w{\left[\%\mathrm{Ti}\right]}_{L}=w{\left[\%\mathrm{Ti}\right]}_{0}{\left[1-(1-2\Omega_{i}k_{\mathrm{Ti}})f_{s}\right]}^{\frac{k_{\mathrm{Ti}}-1}{\left(1-2\Omega_{i}k_{\mathrm{Ti}}\right)}}$$

where *w*[%Ti]_L_ represents the actual mass fraction of Ti in the liquid phase at the solidification front; and *w*[%Ti]_0_ denotes the initial mass fraction of Ti in molten steel.

#### 2.3.2. Evolution of Ti, C, and N Segregation with Increasing Solid Fraction

**Figure 2:**
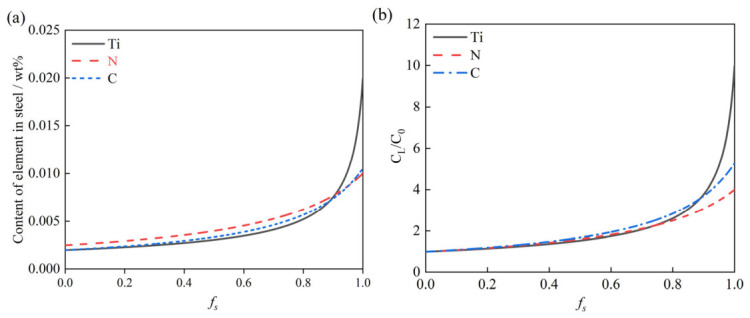
Variation of (**a**) solute content and (**b**) segregation ratio of Ti, C, and N during solidification.

Figure 2 depicts the evolution of concentration and segregation ratios for Ti, C, and N as a function of *f_s_* at a cooling rate of 5 K/s. The results highlight a stark contrast in segregation behavior: Ti exhibits significant solute enrichment, whereas C and N show markedly more moderate variations. Specifically, for an initial Ti content of 0.0020 wt%, the concentration surges to 0.020 wt% at the completion of solidification, yielding a C_L_/C_0_ of 10. In comparison, the enrichment of C and N is less pronounced; for example, initial contents of 0.0025 wt% for N and 0.0020 wt% for C result in terminal concentrations of only 0.010 wt% (C_L_/C_0_ = 4) and 0.0105 wt% (C_L_/C_0_ = 5.26), respectively.

#### 2.3.3. Dependence of Segregation Behavior on Initial Contents of Ti, C, and N 

**Figure 3:**
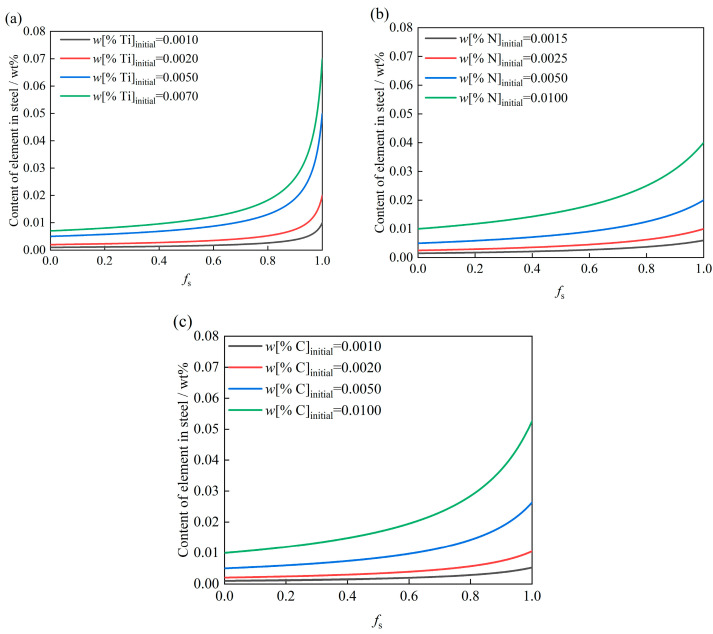
Variation of (**a**) Ti, (**b**) N, and (**c**) C during solidification for different initial contents.

Figure 3 depicts the segregation profiles of Ti, N, and C during solidification at a cooling rate of 5 K/s for varying initial contents. For Ti (Figure 3a), increasing the initial content from 0.0010 to 0.0070 wt% elevates the terminal content from 0.01 to 0.07 wt%. Similarly, for N (Figure 3b), raising the initial content from 0.0015 to 0.01 wt% results in a corresponding increase in the final content from 0.006 to 0.04 wt%. For C (Figure 3c), an increment in initial content from 0.0010 to 0.0100 wt% causes the final content from 0.0053 to 0.0526 wt%.

#### 2.3.4. Effect of Cooling Rate on Solute Segregation of Ti, C, and N

Motivated by the heterogeneous cooling conditions inherent to the thickness direction of thin slabs and guided by prior literature [55], this study focuses on the solidification behavior within ESP, specifically examining cooling rates ranging from 0.1 to 50 K/s.

**Figure 4:**
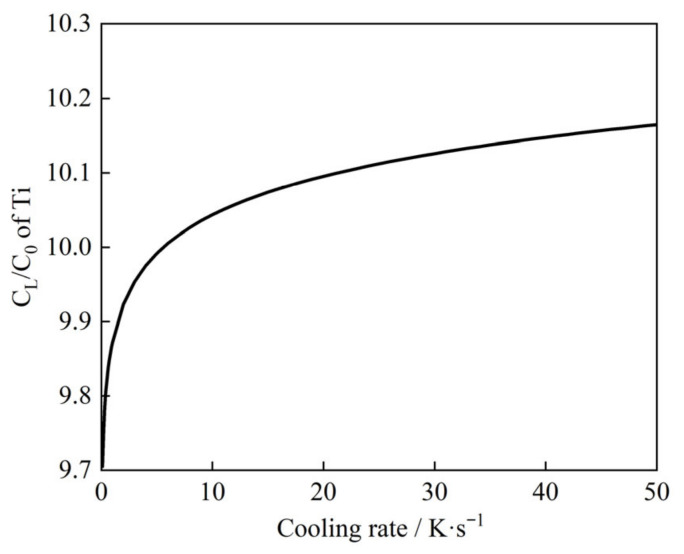
Effect of cooling rate on solute segregation of Ti.

Equations (7) and (8) reveal that while the cooling rate significantly influences the segregation of Ti (due to its slow diffusion), the behavior of (C, N) remains independent of thermal conditions. Figure 4 illustrates the evolution of the Ti segregation ratio (C_L_/C_0_) at the completion of solidification under varying cooling rates. The results demonstrate a positive correlation between cooling rate and Ti segregation severity: as the cooling rate increases from 0.1 to 50 K/s, the C_L_/C_0_ ratio rises monotonically from 9.70 to 10.16.

In summary, the calculation results based on the segregation model indicate that the solid fraction, initial element content, and cooling rate all exert an influence on microsegregation. Specifically, the element content increases with the solid fraction, and higher initial contents lead to correspondingly higher contents at the end of solidification. Furthermore, the C_L_/C_0_ ratio of Ti at the solidification endpoint increases with the cooling rate.

### 2.4. Thermodynamic Condition of Ti(C,N) Precipitation During Solidification

When lg*Q*_Ti(C,N)_ > lg*K*_Ti(C,N)_ [56], the thermodynamic conditions for the precipitation of Ti(C,N) at the solidification front are satisfied. The *Q*_Ti(C,N)_ is defined as follows:

(17) $$Q_{\mathrm{Ti}(C,N)}=w{\left[\%\mathrm{Ti}\right]}_{L}\times w{\left[\%C,\%N\right]}_{L}$$

The temperature at the solidification front *T* is given in Equation (18) [42], which is as follows:

(18) $$T=T_{\mathrm{Fe}}-\frac{T_{\mathrm{Fe}}-T_{L}}{1-f_{s}\times (T_{L}-T_{S})/(T_{\mathrm{Fe}}-T_{S})}$$

The activity coefficients of solute elements Ti and (C,N) at the solidification front are calculated considering local enrichment by Equations (19) and (20), respectively [29,57], which are depicted as follows:

(19) $$\mathrm{lg}f_{\mathrm{Ti}}=\left(\frac{2557}{T}-0.365\right)\times \mathrm{lg}f_{\mathrm{Ti}(1873K)}$$

(20) $$\mathrm{lg}f_{N}=\left(\frac{3280}{T}-0.75\right)\times \mathrm{lg}f_{N(1873K)}$$

**Figure 5:**
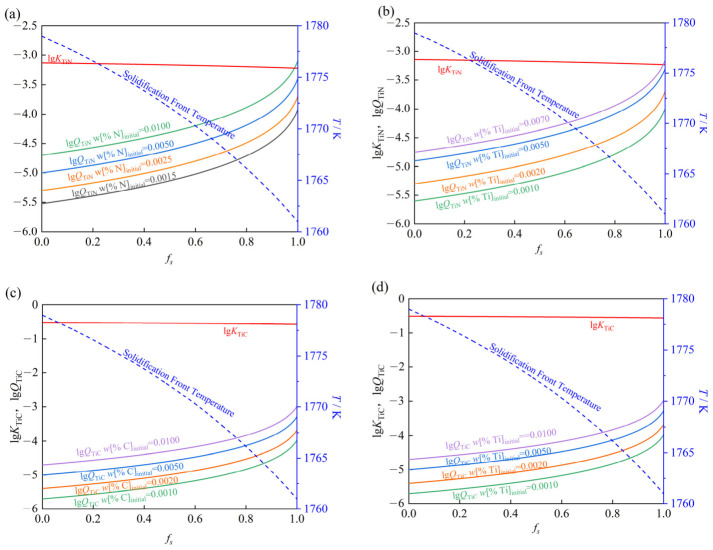
Evolution of (**a**,**b**) Ti–N and (**c**,**d**) Ti–C concentration products as a function of *f_s_* for varying initial Ti, N, and C contents.

The governing parameters were integrated into Equations (7), (8) and (17). The calculated dependencies of *Q*_Ti(C,N)_, *K*_Ti(C,N)_ and *f*_s_ are presented in Figure 5.

Figure 5a depicts the evolution of *Q*_TiN_ and *K*_TiN_ with respect to *f_s_* under varying initial nitrogen contents (fixed *w*[%Ti]_intial_ = 0.0020). While no intersection between the *Q*_TiN_ and *K*_TiN_ curves is observed for initial nitrogen contents of 0.0015, 0.0025, and 0.0050 wt%, an intersection occurs at *f_s_* = 0.9879 for 0.0100 wt% nitrogen. Similarly, Figure 5b presents the results for varying initial titanium contents (fixed *w*[%N]_intial_ = 0.0025). The curves exhibit no intersection for titanium contents up to 0.0050 wt%; however, an intersection is observed at *f_s_* = 0.9929 when the initial titanium content reaches 0.0070 wt%.

Figure 5c,d present analogous analyses for the Ti–C system under varying C (fixed *w*[%Ti]_intial_ = 0.0020) and Ti (fixed *w*[%C] = 0.0020) contents, respectively. Notably, no intersections between lg*Q*_TiC_ and lg*K*_TiC_ occur across the entire investigated compositional range.

The thermodynamic calculations demonstrate that elevated initial concentrations of Ti and N are necessary to fulfill the thermodynamic conditions for TiN precipitation. The thermodynamic basis for TiC precipitation was not fulfilled within the assumed ranges of Ti and C contents. Consequently, reducing the initial contents of Ti, N, and C helps prevent the thermodynamic conditions for TiN and TiC precipitation from being satisfied.

### 2.5. Kinetic Analysis of TiN Inclusion Growth During Solidification

Previous studies [27,40,42,58,59] have shown that TiN inclusions can exist either as isolated particles or as complex inclusions associated with other phases. Given that this study relies on a mathematical modeling approach, the discussion focuses primarily on isolated TiN inclusions in steel.

#### 2.5.1. Kinetic Model of TiN Inclusion Growth

It is when the actual content of titanium and nitrogen in the residual liquid steel reaches equilibrium that TiN inclusions begin to precipitate and grow, as noted above. A diffusion-controlled growth model is employed to characterize the growth behavior of TiN inclusions during the solidification of steel. The model has been widely employed to predict the growth kinetics of TiN in diverse steel grades, including bearing steel and tire cord steel [33,38,42,59]. The current model excludes particle–particle interaction, interface pushing/engulfment, non-spherical morphology, and the possible formation of complex inclusions near the end of solidification.

The model is based on the following assumptions:

(1) inclusions are spherical;
(2) the system operates under steady-state diffusion conditions;
(3) inclusions grow independently without mutual interaction.

Given the microsegregation of Ti and N, TiN nucleation and growth initiate once the local solute product at the solid–liquid interface satisfies the thermodynamic equilibrium criterion. The rapid diffusion of nitrogen at the solidification front is the rate-limiting step, hindering the precipitation and growth of titanium inclusions [27,38,40,59]. Consequently, the diffusive flux of nitrogen is defined by Equation (21) as follows:

(21) $$J=\frac{D_{N}}{r}\frac{\rho_{m}}{100M_{N}}(w{\left[\%N\right]}_{L}-w{\left[\%N\right]}_{e})$$

where *J* is the diffusion flux of N (mol/m^2^·s), *r* is the radius of TiN (cm), D_N_ is the diffusion coefficient of N, *M*_N_ represents the molar mass of N (14 g/mol), *ρ*_m_ is the density of molten steel (7.07 g/cm^3^), and *w*[%N]_L_ and *w*[%N]_e_ are the mass fractions of N at the solidification front and in the equilibrium state, respectively.

The correlation between the nitrogen diffusive flux and the evolution of the TiN inclusion radius is described by Equation (22) as follows:

(22) $$4\pi r^{2}M_{\mathrm{TiN}}J\Delta t=\frac{4}{3}\pi \rho_{\mathrm{TiN}}[{\left(r+\Delta r\right)}^{3}-r^{3})$$

where *M*_TiN_ is the molar mass of TiN (62 g/mol), and *ρ*_TiN_ is the density of TiN (5.43 g/cm^3^).

It can be obtained from Equations (21) and (22) that in the molten steel solidification front, based on the N content of the chemical composition of high-grade non-oriented silicon steel, the theoretical precipitation size *r* of TiN at the solidification front [31,34] can be calculated using Equation (23), namely:

(23) $$r\frac{dr}{dt}=\frac{M_{\mathrm{TiN}}}{100M_{\mathrm{Fe}}}\times \frac{\rho_{\mathrm{Fe}}}{\rho_{\mathrm{TiN}}}\times D_{N}\times (w{\left[\%N\right]}_{L}-w{\left[\%N\right]}_{e})$$

Integrating Equation (23), the following can be obtained:

(24) $$r=\sqrt{\frac{M_{\mathrm{TiN}}}{50M_{\mathrm{Fe}}}\times \frac{\rho_{\mathrm{Fe}}}{\rho_{\mathrm{TiN}}}\times D_{N}\times {\left(w{\left[\%N\right]}_{L}-w[\%N]\right)}_{e})\times \tau }$$

#### 2.5.2. Effect of Cooling Rate on Inclusion Growth

**Figure 6:**
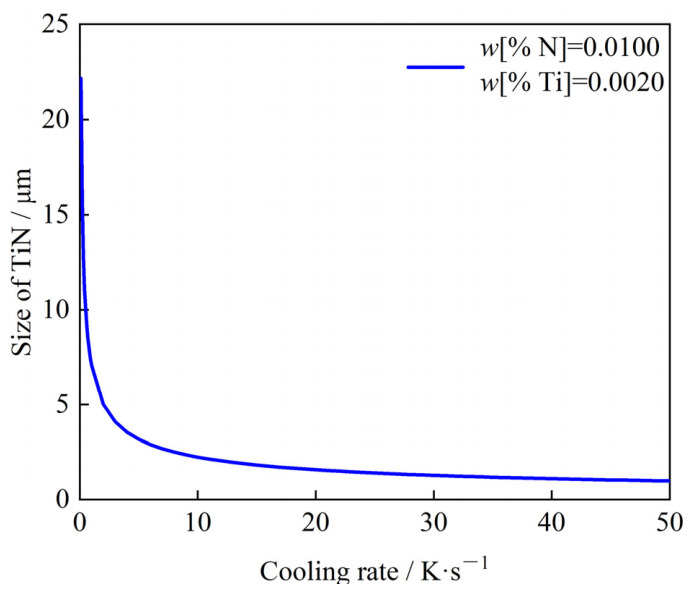
Effect of cooling rate on the size of TiN inclusion at the end of solidification.

Figure 6 illustrates the effect of cooling rate on the size of TiN inclusions at the end of solidification. The results indicate that with fixed initial concentrations of Ti and N, a higher cooling rate refines the final TiN size. While fine inclusions are generally beneficial for refining the solidification structure and improving steel strength, studies on high-grade non-oriented silicon steel [16,17,18,19,20,21,22] have shown that excessively fine TiN (<1 μm) can adversely affect magnetic properties by pinning domain walls. As shown in Figure 6, the model calculations suggest that the size of TiN inclusions will be less than 1 μm when the cooling rate exceeds 19 K/s.

#### 2.5.3. Effect of Initial Solute Content on Inclusion Growth

**Figure 7:**
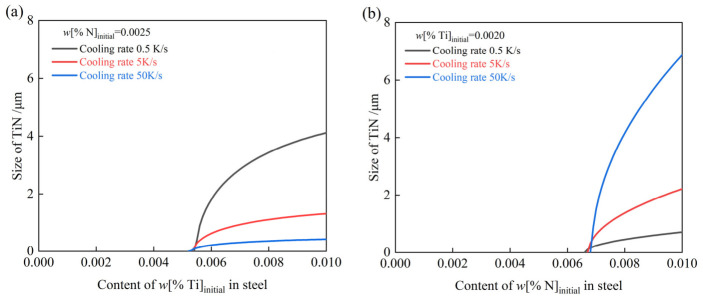
Effect of varying (**a**) *w*[%Ti]_intial_ and (**b**) *w*[%N]_intial_ on the size of TiN inclusions.

Figure 7a depicts the effect of varying *w*[%Ti]_intial_ on the size of TiN inclusions at the end of solidification with a fixed *w*[%N]_intial_ = 0.0025. Conversely, Figure 7b illustrates the influence of varying *w*[%N]_intial_ with a fixed *w*[%Ti]_intial_ = 0.0020, considering cooling rates of 0.5 K/s (slab center), 5 K/s (average), and 50 K/s (slab surface). Figure 7a,b exhibit similar trends: at a constant cooling rate, the inclusion size increases with higher initial solute content; whereas at a fixed solute content, higher cooling rates result in smaller inclusions. Furthermore, a higher cooling rate requires a higher initial N or Ti content to initiate TiN growth. The critical threshold for Ti is 0.0052–0.0054 wt%, while that for N is 0.0066–0.0068 wt%.

#### 2.5.4. Effect of Solidification Fraction on Inclusion Growth

**Figure 8:**
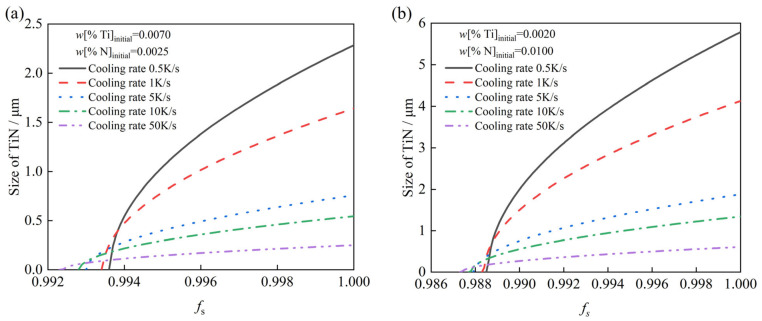
Effect of *f_s_* and cooling rate on TiN inclusion size during solidification. (**a**) *w*[%Ti]_intial_ = 0.0070, *w*[%N]_intial_= 0.0025; (**b**) *w*[%Ti]_intial_ = 0.0020, *w*[%N]_intial_ = 0.0100.

Figure 8a,b illustrate the evolution of TiN inclusion size as a function of the *f_s_* in two distinct composition regions: high Ti/low N (*w*[%Ti]_intial_ = 0.0070, *w*[%N]_intia_ = 0.0025) and low Ti/high N (*w*[%Ti]_intial_ = 0.0020, *w*[%N]_intial_ = 0.0100). Five different cooling rates were considered to comprehensively evaluate their influence. The model calculations indicate that at a constant cooling rate the TiN inclusion size increases with *f_s_*; conversely, at any given *f_s_*, a higher cooling rate results in smaller inclusions. Furthermore, a higher cooling rate leads to a lower *f_s_* value for the onset of TiN precipitation. The primary reason is that a high cooling rate shortens the local solidification time, thereby reducing the growth time available for TiN inclusions and resulting in finer particles.

Based on the model calculations above, the factors influencing TiN inclusion size mainly include cooling rate, initial Ti and N solute content, and solid fraction. Among these, the solid fraction is intrinsic to the solidification process and difficult to manipulate through process adjustments. Therefore, to avoid the formation of fine inclusions that adversely affect the magnetic properties of high-grade non-oriented silicon steel, it is feasible to reduce the cooling rate and lower the Ti and N content in the steel.

In ESP, there exists a critical contradiction: although reducing the cooling rate facilitates the formation of larger inclusions to mitigate magnetic property degradation, it fundamentally compromises the inherent high-speed production advantage of ESP, thereby limiting productivity. Thus, optimizing the solidification cooling rate is largely impractical.

Previous studies [60,61,62] indicate that the nitrogen content in liquid steel primarily originates from raw materials and additives, as well as air reoxidation or absorption due to poor sealing. To achieve effective denitrification, several key measures are required: reducing nitrogen input from raw materials, increasing the hot metal ratio, and intensifying decarburization reactions in the converter or EAF. Furthermore, the implementation of vacuum degassing (e.g., RH and VD) combined with protective casting has proven effective in lowering nitrogen content. However, further reducing nitrogen content during the final casting stage remains a formidable challenge. In contrast, research regarding the source tracing and control mechanisms of titanium in steel is relatively limited. Therefore, systematically tracing the sources of titanium in molten steel and formulating effective control strategies is of critical importance.

## 3. Titanium Sources and Process Control Strategies

### 3.1. The Sources of Titanium

Titanium sources in high-grade non-oriented silicon steel are categorized into two types: (1) primary Ti, derived directly from raw materials (iron, scrap steel) and alloy additions; (2) secondary Ti, stemming from slag and refractory linings. The latter involves the decomposition of TiO_2_ in the molten steel or its reduction by deoxidizers, which results in the liberation of metallic titanium into the melt.

#### 3.1.1. Primary Ti

Primary Ti originates predominantly from metallic inputs, specifically iron, scrap steel, and alloy additives. The mass fraction of Ti introduced by these sources is quantified by Equation (25), which is as follows:

(25) $$w{\left[\%\mathrm{Ti}\right]}_{\mathrm{total}}=\sum w{\left[\%\mathrm{Ti}\right]}_{s}=\sum \frac{\beta_{s}\times M_{s}\times w{\left[\%\mathrm{Ti}\right]}_{s}^{0}}{M_{\mathrm{Steel}}}$$

where *w*[%Ti]*_s_* denotes the Ti mass fraction contributed by source *s* (iron, scrap steel, or alloy); *β_s_* represents the recovery yield; *M_s_* is the mass addition; ${w[\%Ti]}_{s}^{0}$ is the initial Ti mass fraction; and *M*_Steel_ is the total mass of the molten steel bath.

As indicated by Equation (25), the total amount of titanium (Ti) introduced into the molten steel via iron, scrap steel, and various alloys is governed by three key factors: (1) the actual recovery yield of Ti in the molten steel, which directly determines the effective proportion of Ti entering the melt; (2) the actual addition amounts of iron, scrap steel, and alloys, serving as the fundamental basis for the total Ti input; (3) the initial mass fraction of Ti in these materials, reflecting the intrinsic Ti level of the raw materials.

#### 3.1.2. Secondary Ti

Beyond primary inputs, secondary Ti constitutes a critical contributor to excessive Ti levels in molten steel. This fraction originates primarily from the reduction of titanium dioxide (TiO_2_) present in slags and refractory linings, which releases dissolved [Ti] into the melt. TiO_2_ sources in slag are diverse, encompassing converter slag carryover, refinement synthetic slags, and auxiliary fluxes (e.g., limestone, fluorite). Refractory-derived TiO_2_ arises from equipment throughout the smelting chain, including converter and ladle linings, RH immersion tubes, tundish linings, and functional components such as nozzles and stoppers.

(1) Effect of Slag Carryover and TiO_2_ Content of Slag

During converter (Basic Oxygen Furnace, BOF) smelting, Ti from iron and scrap oxidizes form TiO_2_ and transfer into the converter slag. During tapping, a portion of this slag is inevitably entrained into the subsequent refining stage [63]. Therefore, the amount of BOF slag carryover significantly affects the titanium increment during the RH refining process. Based on the principle of mass balance (Equation (26)), the quantitative relationship between the BOF slag carryover (*W*_BOF_(kg/t)) and the partial RH titanium increment (ΔTi_BOF slag_) caused by this slag can be derived as Equation (27). To isolate the dependence on key variables, the following assumptions are adopted: (1) the initial RH slag is TiO_2_-free; (2) the mass fraction of TiO_2_ in the BOF slag is denoted as *T*_BOF_(%); (3) the RH slag addition (*W*_RH_(kg/t)) is standardized to 15 kg/t; (4) the mass fraction of residual titanium dioxide in the RH slag (*T*_RH_(%)) is fixed at 0.05% [63,64]. The equations are as follows:

(26) $$W_{\mathrm{BOF}}\times T_{\mathrm{BOF}}\times \frac{48}{80}=W_{\mathrm{RH}}\times T_{\mathrm{RH}}\times \frac{48}{80}+\Delta {\left[\%\mathrm{Ti}\right]}_{\mathrm{BOF}\;\mathrm{slag}}\times 10^{-3}$$

(27) $$\Delta {\left[\%\mathrm{Ti}\right]}_{\mathrm{BOF}\;\mathrm{slag}}=(W_{\mathrm{BOF}}\times T_{\mathrm{BOF}}\times \frac{48}{80}-0.0045)\times 10^{3}$$

**Figure 9:**
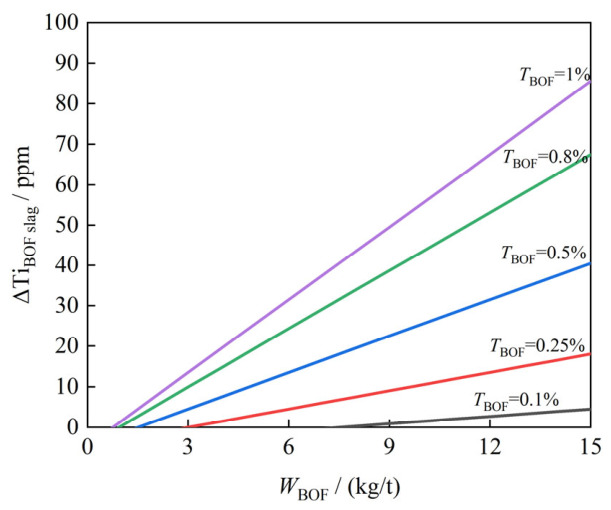
Effect of BOF slag carryover and slag TiO_2_ content on ΔTi_BOF slag_ of RH.

Figure 9 illustrates the combined effect of BOF slag carryover and its TiO_2_ content on the theoretical calculation of ΔTi_BOF slag_ in RH-refined steel. The data reveals a significant positive correlation: as both parameters increase, ΔTi_BOF slag_ exhibits a sharp upward trend. Therefore, to prevent excessive titanium accumulation, strict control of these two parameters is crucial for the production of high-grade non-oriented silicon steel. According to the theoretical calculations, when the TiO_2_ content in the slag is 0.5%, a carryover amount exceeding 9 kg/t will result in a ΔTi_BOF slag_ greater than 20 ppm, which exceeds the compositional limit for this steel grade.

(2) Effect of TiO_2_

During the subsequent refining stage, the decomposition of TiO_2_ transferring from the refining slag into the molten steel is governed by Equation (28), which is as follows:

(28) $${\mathrm{TiO}}_{2}(s)=\left[\mathrm{Ti}\right]+2[O]\;\;\Delta G_{{\mathrm{TiO}}_{2}}^{\theta }=-228.33T+681600$$

(29) $$K_{{\mathrm{TiO}}_{2}}=\frac{a_{{\mathrm{TiO}}_{2}}}{f_{\left[\mathrm{Ti}\right]}\times w[\%\mathrm{Ti}]\times f_{\left[O\right]}^{2}\times w{\left[\%O\right]}^{2}}$$

(30) $$w[\%\mathrm{Ti}]=\frac{a_{{\mathrm{TiO}}_{2}}\times e^{-\frac{\Delta G_{{\mathrm{TiO}}_{2}}^{\theta }}{\mathrm{RT}}}}{f_{\left[\mathrm{Ti}\right]}\times f_{\left[O\right]}^{2}\times w{\left[\%O\right]}^{2}}$$

The oxygen content was derived based on Ti-O thermodynamic equilibrium conditions. This calculation serves to evaluate the impact of theoretical oxygen activity on the decomposition reaction, thereby providing guiding measures to control the secondary [Ti] obtained from TiO_2_ decomposition in steel.

**Figure 10:**
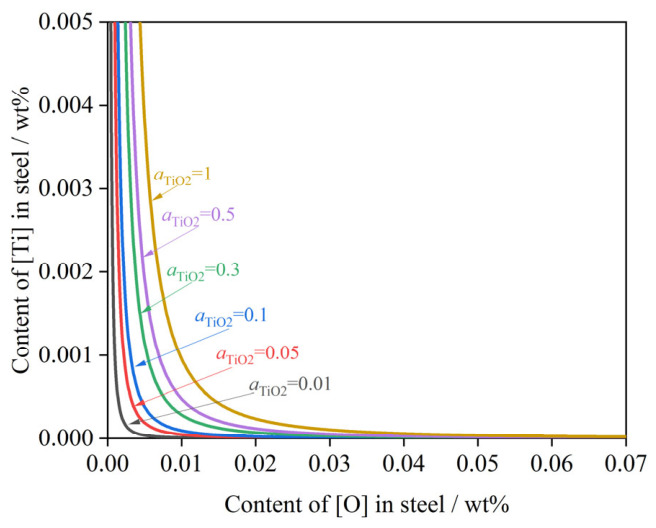
Ti-O equilibrium phase diagram of molten steel at 1873 K.

Figure 10 illustrates the Ti–O equilibrium in molten steel at a constant temperature of 1873 K, with calculated $a_{{TiO}_{2}}$ values of 0.01, 0.05, 0.1, 0.3, 0.5, and 1.0. The theoretical calculations indicate a significant negative correlation between [O] and [Ti] under fixed thermodynamic conditions: as the [O] content increases, the equilibrium [Ti] content decreases monotonically. Furthermore, lower $a_{{TiO}_{2}}$ values shift the equilibrium curves downward, resulting in lower equilibrium [Ti] content at a given [O] content.

**Figure 11:**
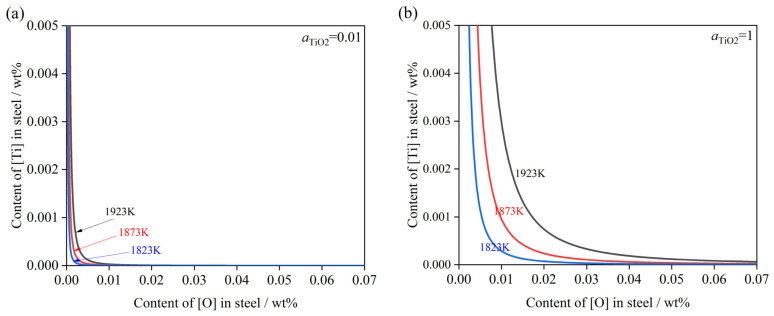
Ti-O equilibrium phase diagram of molten steel. (**a**) $a_{{TiO}_{2}}$ = 0.01; (**b**) $a_{{TiO}_{2}}$ = 1.

Figure 11 shows the Ti–O equilibrium in molten steel at temperatures of 1823 K, 1873 K, and 1923 K, with specific $a_{{TiO}_{2}}$ values of 0.01 (Figure 11a) and 0.1 (Figure 11b). The calculation results for both cases indicate that when $a_{{TiO}_{2}}$ is constant, increasing the temperature promotes the decomposition reaction of TiO_2_. Consequently, a higher [O] content is required to achieve the thermodynamic equilibrium required for obtaining low [Ti] levels in the steel.

(3) Effect of [Al] on [Ti]

High-grade non-oriented silicon steel is distinguished by its high aluminum and silicon contents coupled with low carbon levels. Aluminum addition during refining is essential for deoxidation and composition control. However, an increase in dissolved aluminum [Al] triggers the reduction of TiO_2_ from the slag into the melt, resulting in a rebound of the total titanium content. The governing chemical reaction transpiring at the steel–slag interface between dissolved [Al] in the melt and TiO_2_ in the slag is presented below [65]:

(31) $$\frac{2}{3}\left[\mathrm{Al}\right]+\frac{1}{2}{\mathrm{TiO}}_{2}\left(s\right)=\frac{1}{3}{\mathrm{Al}}_{2}O_{3}\left(s\right)+\frac{1}{2}\left[\mathrm{Ti}\right]\;\Delta G_{{\mathrm{TiO}}_{2}/{\mathrm{Al}}_{2}O_{3}}^{\theta }=-60905+15.08T$$

(32) $$K_{{\mathrm{TiO}}_{2}/{\mathrm{Al}}_{2}O_{3}}=\frac{{a_{\left[\mathrm{Ti}\right]}}^{\frac{1}{2}}\times {a_{\left({\mathrm{Al}}_{2}O_{3}\right)}}^{\frac{1}{3}}}{{a_{\left[\mathrm{Al}\right]}}^{\frac{2}{3}}\times {a_{\left({\mathrm{TiO}}_{2}\right)}}^{\frac{1}{2}}}=\frac{{f_{\mathrm{Ti}}}^{\frac{1}{2}}\times w{\left[\%\mathrm{Ti}\right]}^{\frac{1}{2}}\times {a_{\left({\mathrm{Al}}_{2}O_{3}\right)}}^{\frac{1}{3}}}{{f_{\mathrm{Al}}}^{\frac{2}{3}}\times w{\left[\%\mathrm{Al}\right]}^{\frac{2}{3}}\times {a_{\left({\mathrm{TiO}}_{2}\right)}}^{\frac{1}{2}}}$$

Simplified formula:

(33) $$w([\%\mathrm{Ti}])=\frac{(e^{-\frac{\Delta G^{\theta }}{\mathrm{RT}}}{)}^{2}\times {f_{\mathrm{Al}}}^{\frac{4}{3}}\times w{\left[\%\mathrm{Al}\right]}^{\frac{4}{3}}\times a_{\left({\mathrm{TiO}}_{2}\right)}}{f_{\mathrm{Ti}}\times {a_{\left({\mathrm{Al}}_{2}O_{3}\right)}}^{\frac{2}{3}}}=\frac{A\times w{[\%\mathrm{Al}]}^{\frac{4}{3}}}{m^{2}}$$

wherein for $A=\frac{(e^{-\frac{\Delta G^{\theta }}{RT}}{)}^{2}\times {f_{\mathrm{Al}}}^{\frac{4}{3}}}{f_{\mathrm{Ti}}}$, *A* is a constant at a certain temperature; and $m=\frac{{a_{\left({\mathrm{Al}}_{2}O_{3}\right)}}^{\frac{1}{3}}}{{a_{\left({\mathrm{TiO}}_{2}\right)}}^{\frac{1}{2}}}$, *m* represents the activity ratio of Al_2_O_3_ to TiO_2_ in the slag, serving as an indicator of the thermodynamic driving force for the reduction of (TiO_2_) by [Al] in the steel.

Given the typical composition of high-grade non-oriented silicon steel presented in Table 1, Equation (33) simplifies to the following:

(34) $$w[\%\mathrm{Ti}]=\frac{\begin{array}{r} 75.1332 \end{array}\times w{\left[\%\mathrm{Al}\right]}^{\frac{4}{3}}}{m^{2}}$$

**Figure 12:**
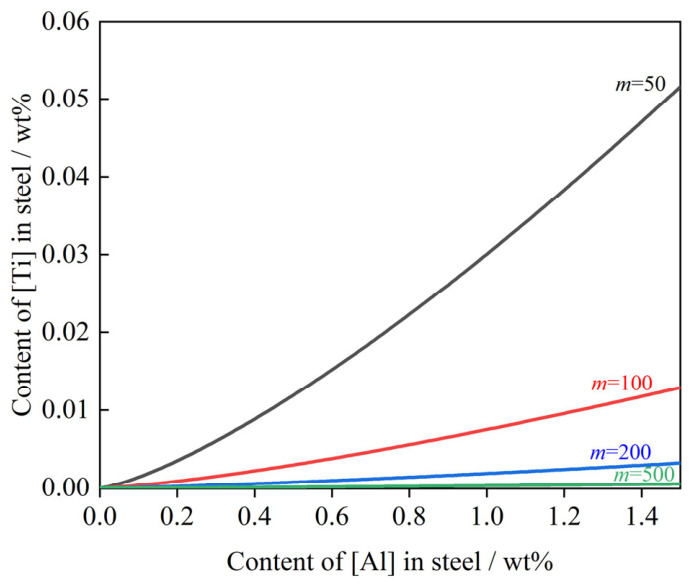
Relationship between *w*[%Ti] and *w*[%Al] at the steel–slag interface at 1873 K.

Figure 12 illustrates the relationship between dissolved titanium [Ti] and aluminum [Al] in molten steel at the steel–slag interface at 1873 K. The results indicate that at a constant activity ratio *m*, an increase in [Al] leads to a simultaneous rise in [Ti]; that is, a higher [Al] content results in a higher [Ti] level. However, as the value of *m* increases, the sensitivity of [Ti] to changes in [Al] decreases. This implies that increasing the $a_{{{Al}_{2}O}_{3}}$ and decreasing the $a_{{TiO}_{2}}$ at the interface helps mitigate the impact of [Al] on [Ti] in the molten steel.

### 3.2. Control Strategies

Based on the analysis of titanium sources mentioned above, to reduce the dissolved [Ti] content in steel, corresponding control measures must be taken regarding its sources: First, strict control of the initial Ti content in raw materials (iron, scrap) and alloys is necessary to minimize primary Ti input. Second, reducing the TiO_2_ content in slag or refractories is essential to decrease the generation of secondary Ti.

#### 3.2.1. Control Strategies of Primary Ti

To precisely regulate primary Ti input: (1) Optimize blast furnace inputs: Utilize low-Ti iron ores to minimize the intrinsic Ti content of iron. (2) Implement strategic scrap selection: Prioritize low-Ti scrap grades and optimize the iron-to-scrap ratio (despite the typically elevated Ti levels in iron compared to scrap, an iron-to-scrap ratio of at least 85% is required to strictly regulate nitrogen content). (3) Enforce strict alloy qualification: Screen and select ultra-low-Ti alloys (e.g., Fe-Si, Al). (4) Modulate recovery kinetics: Optimize smelting conditions to suppress the recovery yield *β_s_* by promoting the oxidation of dissolved [Ti] into stable oxide complexes in the slag.

#### 3.2.2. Control Strategies of Secondary Ti

(1) Reduction in Slag Carryover

Based on the calculation results regarding BOF slag in Section 3.1.2, the primary measure is to reduce the amount of slag carryover during tapping. This can be achieved by employing slag blocking methods, such as slide gates or slag stoppers. Secondly, for heats with excessive slag carryover, immediate slag skimming after tapping is recommended. These measures will help suppress the source of secondary titanium generation.

(2) Reduction in secondary Ti Content in Steel

Guided by the analysis in Section 3.1.2, the following measures are proposed to lower secondary Ti levels in steel: (1) Reduce the activity of TiO_2_ in the slag. This can be achieved by lowering the TiO_2_ content in BOF slag carryover and adding low-TiO_2_ auxiliary materials to dilute the TiO_2_ in RH refining slag. (2) Appropriately increasing the [O] content prior to BOF tapping and reducing the refining temperature serve to suppress the decomposition reaction of titanium dioxide at the steel–slag interface during RH refining. (3) On the premise of meeting the performance requirements of high-grade non-oriented silicon steel, reduce dissolved [Al] in the steel and appropriately increase Al_2_O_3_ content in the slag to suppress the reduction reaction of TiO_2_ by [Al].

## 4. Conclusions

Based on an investigation into the formation mechanism of Ti(C,N) inclusions and titanium control strategies in high-grade non-oriented silicon steel, the following conclusions are drawn from the calculation results:

1. In the high-grade non-oriented silicon steel under study, Ti(C,N) inclusions do not precipitate at temperatures above the liquidus (1779 K).
2. During solidification, microsegregation enriches Ti, C, and N. The ion product of TiC remains undersaturated, preventing precipitation, and the ion product of TiN exceeds its solubility limit, driving the precipitation and growth of TiN inclusions in the final solidification stage—findings valid within the present composition window and modeling framework.
3. The size of TiN inclusions is primarily governed by the cooling rate and initial Ti/N contents. A higher cooling rate yields smaller inclusions, while reducing the Ti/N content effectively suppresses TiN precipitation.
4. Titanium sources in this steel include primary Ti from raw materials (e.g., iron, scrap) and alloy additives, as well as secondary Ti generated from the decomposition or reduction of TiO_2_ in slag and refractories. Mitigating [Ti] content requires a dual approach: (1) strictly limiting primary Ti input from raw materials and alloys; (2) effectively suppressing secondary Ti formation by curtailing BOF slag carryover, reducing slag TiO_2_ activity, lowering the refining temperature, and controlling [Al] content.

## Figures and Tables

**Table 1:** Chemical composition (wt%).

Element	C	Si	Mn	P	S	Als	Ti	N	Fe
Range	≤0.003	3.1~3.25	0.25~0.3	≤0.02	≤0.0025	0.85~1.0	≤0.0020	≤0.0025	Balance
Target	0.0020	3.2	0.264	0.013	0.0020	0.97	0.0020	0.0025	Balance

**Table 2:** Interaction coefficients between components at 1873 K [42,49,50].

$e_{i}^{j}$	C	Si	Mn	P	Al	N	Ti	S	Ca
Ti	−0.165	0.05	0.0043	−0.0064	0.004	−1.8	0.013	−0.11	−0.157
N	0.13	0.047	−0.021	0.045	−0.028	0	−0.53	0.007	
C	0.14	0.08	−0.012	0.051	0.043	0.11		0.046	
Al	0.091	0.0056	0.035	0.033	0.045	−0.058	0.004	0.03	−0.05
O	−0.45	−0.131	−0.021	−0.07	−3.9	0.057	−1.8	−0.133	−515

**Table 3:** Diffusion coefficients and equilibrium partition coefficients of Ti, C, and N in δ-Fe [40].

Element	*k* of δ-Fe	*D_S_*/(cm^2^·s^−1^) of δ-Fe
Ti	0.38	3.15 exp(−247,693/RT)
N	0.25	0.008 exp(−79,078/RT)
C	0.19	0.00127 exp(−81,379/RT)

## Data Availability

The original contributions presented in this study are included in the article. Further inquiries can be directed to the corresponding author.
